# The End of the Line for Hookworm? An Update on Vaccine Development

**DOI:** 10.1371/journal.pmed.0020327

**Published:** 2005-10-04

**Authors:** Eileen Devaney

## Abstract

Gut-associated molecules of human hookworms have been the focus of recent interest as potential vaccine candidates.

Human hookworms are parasitic nematodes infecting about 700 million individuals, largely in tropical regions of the world [1]. In endemic areas, most infected people carry a mixed worm burden, including Ascaris lumbricoides (roundworms), Trichuris trichuria (whipworms), and Ancylostoma duodenale and/or Necator americanus (both hookworms). Of these soil-transmitted helminths, hookworms are the most pathogenic because of their propensity to feed on blood, resulting in anaemia, particularly in those with low iron reserves such as children and women of reproductive age.

## The Pathogenesis of Hookworm Infection

Hookworms' blood-feeding (hematophagous) habits cause pathology in humans and animals. The worms attach to the wall of the small intestine using their mouthparts and feed on blood from ruptured capillaries. Each female worm is estimated to ingest a minimal 0.1 ml of blood per day. However, actual blood loss can be significantly greater; the worms change their feeding sites several times a day, and the secretion of anti-coagulants means that the vacated sites continue to bleed, contributing greatly to blood loss.

Hookworms do not kill, but they can cause subclinical disease, most notably anaemia and impaired physical and cognitive development in children. As hookworm infection is associated with low socioeconomic status, it adds significantly to the burden of disease in such areas [1].

## Natural Infection Elicits Poor Immunity

Hookworms have a simple life cycle in which the third-stage larvae (L3) infect humans, generally by skin penetration, although some species are also infective via oral ingestion. The parasites enter the bloodstream and migrate to the lungs; from there, they are coughed and swallowed to the small intestine (Figure 1). The adult parasites mature in the intestine, and following mating, the female worm produces many thousands of eggs that pass out in the faeces and develop on the ground to infective L3.

**Figure 1:**
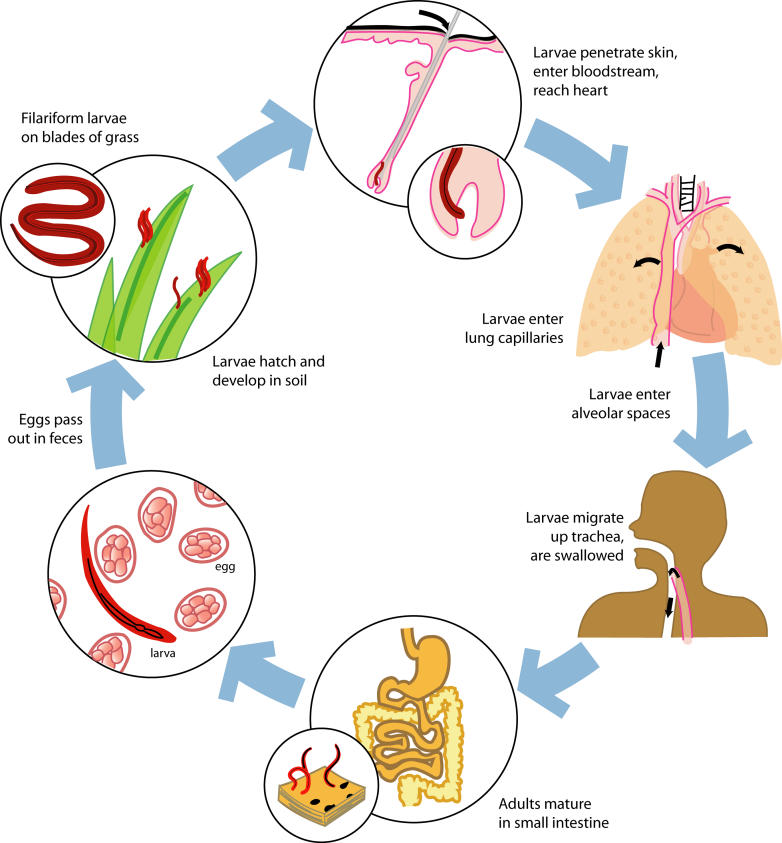
Life Cycle of the Human Hookworm N. americanus (Illustration: Sapna Khandwala, reproduced from [13])

Hookworms can be treated using anthelmintic drugs such as albendazole, but treated people soon become reinfected. Additionally, recent epidemiological studies from China and Brazil show the highest worm burden and the highest prevalence of infection in the elderly [2], contrasting with the intensity/prevalence curves for other soil-transmitted helminths, which typically peak in mid-to-late childhood. These data suggest that under natural conditions of exposure, little immunity is evoked. Given this immuno-epidemiological picture, developing a vaccine is a significant challenge.

## The Search for a Vaccine

Recent studies suggest that the hematophagous lifestyle of hookworms may prove their downfall. Hookworms are armed with an array of molecules that are essential for blood feeding and digestion; these include anticoagulants and a variety of proteases that digest haemoglobin (Hb) and other serum proteins. In a new article in *PLoS Medicine*, Loukas et al. [3] now describe a vaccination schedule using one such protease—an aspartic haemoglobinase—from the hookworm Ancylostoma caninum (*Ac*-APR-1). In their study, the schedule protected against blood loss in an animal model of hookworm infection.

As with other parasitic nematodes, hookworms are complex multicellular organisms that have evolved an array of mechanisms for suppressing or avoiding host immune responses. The only commercially available vaccine against a parasitic nematode is Huskvac (an oral lungworm vaccine for calves), a preparation of radiation-attenuated L3 of Dictyocaulus viviparus, which protects against parasitic bronchitis [4]. In the 1960s, a similar approach was adopted to controlling hookworm infection in dogs using irradiated L3 of A. caninum [5]. While this vaccine was efficacious, it was a commercial failure. However, these older vaccines established the principle that protective immunity can be elicited. The challenge now is to identify protective antigens and present them to the immune system in an appropriate manner.

## Proteases as Potential Vaccine Candidates

Hookworms express a range of proteases, including cysteine, aspartic, and metallo-proteases, several of which have been characterised in detail. These enzymes are localised to the brush border of the worm intestine and have been shown to function in a multi-enzyme cascade to digest Hb and other serum proteins [6]. Some of these molecules show an exquisite specificity; for example, *Na*-APR-2, an aspartic protease from N. americanus, cleaves Hb from the permissive host (human) with twice the efficiency of Hb from a non-permissive host (dog) [7].

These gut-associated molecules of the parasite have been the focus of recent interest as potential vaccine candidates. The rationale behind this approach is that the induction of antibodies to molecules that function in parasite feeding will neutralise their activity and effectively “starve” the worm. A similar approach has previously been trialled against nematode parasites of livestock such as Haemonchus contortus [8], and some important lessons have been learned. One of these is the requirement for expression of recombinant molecules in a eukaryotic system [9]. In general, bacterial-expressed antigens do not stimulate protection, presumably because they are improperly folded and/or modified and catalytically inactive.

Under the auspices of the Human Hookworm Vaccine Initiative and the Sabin Vaccine Institute (http://www.sabin.org/hookworm.htm), which supported Loukas and colleagues' study [3], the drive to develop a hookworm vaccine has gained significant momentum. A number of candidate antigens have been tested in the A. caninum dog model with varying degrees of success. Ideally, vaccination would protect against infection with L3. The same team of researchers have previously identified one such secreted molecule of the L3 of N. americanus, *Na*-ASP-2 (for abundant secreted protein-2), and have shown it to partially protect dogs against infection [10]. Phase 1 safety trials are now underway with this antigen.

Other candidate molecules are the cysteine and aspartic haemoglobinases from the adult worm. In another study by Loukas and colleagues, vaccination with a catalytically active cathepsin-B-like protease from A. caninum (*Ac*-CP-2) produced in the yeast Pischia pastoris resulted in worms that were stunted and produced fewer eggs but did not produce a reduction in number of worms or protect against anaemia [11]. The current study from the same group tested an active aspartic haemoglobinase (*Ac*-APR-1) in the same model [3]. A modest reduction in worm burden was observed in immunised animals, but a highly significant reduction in worm fecundity was observed (up to 85% reduction in mean egg output between vaccinated and adjuvant-only controls), emphasising the nutritional demand of the parasite for egg laying and presumably reflecting an accumulation of neutralising antibodies. Most importantly, four of five vaccinated animals showed a reduction in Hb loss. Thus *Ac*-APR-1 could represent a pathology-limiting component of a future multivalent vaccine [3]. In addition, by restricting worm fecundity it would, in essence, also act as a “transmission blocking” vaccine.

## The Challenges Ahead

Despite these achievements, significant challenges remain, such as the selection of appropriate adjuvants for use in humans, the production of the vaccine at low cost and high yield, its distribution in the tropics, and the possible requirement to de-worm individuals prior to vaccination. However, the Human Hookworm Vaccine Initiative is progressing on many of these fronts and is an excellent example of what can be achieved with proper funding and good collaborations. It has the potential to become a 21st-century paradigm for a control programme aimed at a neglected tropical disease and should also provide renewed impetus to control programmes aimed at vaccine development against other hematophagous helminths of humans and domestic animals.

Finally, it is somewhat ironic to note that as attempts are made to eradicate worm infection in tropical regions of the world, worms are being used in the developed countries to regulate pathogenic proinflammatory immune responses. For example, patients with ulcerative colitis have shown an encouraging amelioration of pathology following infection with the pig whipworm Trichuris suis [12]. These studies demonstrate the capacity of helminth parasites to induce regulatory immune networks in their hosts, and they emphasise the scale of the challenge facing the development of vaccines against worms.

## Abbreviations

Hb: haemoglobin
L3: third-stage larvae
